# Multiple roles of business for climate action

**DOI:** 10.1016/j.isci.2025.114059

**Published:** 2025-11-19

**Authors:** Sam Hampton, Andrea Byfuglien, Will Eadson, Briony Latter, Ben Hardy-Jones, Katherine Sugar, Hannah Bamford, Richard Blundel

**Affiliations:** 1 Environmental Change Institute, School of Geography and Environment, University of Oxford, South Parks Rd, Oxford OX1 3QY, UK; 2 Department of Psychology, University of Bath, 10 West, Claverton Down, Bath BA2 7AY, UK; 3 Sheffield Hallam University, Howard Street, Sheffield S1 1WB, UK; 4 Open University Business School, Walton Hall, Kents Hill, Milton Keynes MK7 6AA, UK; 5 School of Psychology, Cardiff University, Tower Building, 70 Park Place, Cardiff CF10 3AT, UK; 6 School of Environment, Education and Development, University of Manchester, Manchester M13 9PL, UK

**Keywords:** Climatology, Social sciences, Business

## Abstract

Businesses play a critical role in the fight against climate change. While some businesses—both high-profile and less well-known—are making significant strides, most companies are not sufficiently decarbonizing their operations or contributing toward the broader social and economic changes needed to meet climate goals. In response to scrutiny over “greenwashing”, many businesses are becoming cautious about communicating sustainability efforts, a phenomenon known as “greenhushing”. This article aims to mainstream business climate action by introducing a new framework that outlines the roles businesses can adopt in addressing climate change. Based on an interdisciplinary literature review and empirical research from the UK, we identify five roles for businesses. While much focus has been on businesses as energy *consumers* and *enablers* of low-carbon products, we highlight three additional roles: *influencer*, *citizen*, and *investor*. This framework serves as a heuristic tool for researchers, policymakers, and business leaders seeking to promote climate action.

## Graphical abstract

**Figure:**
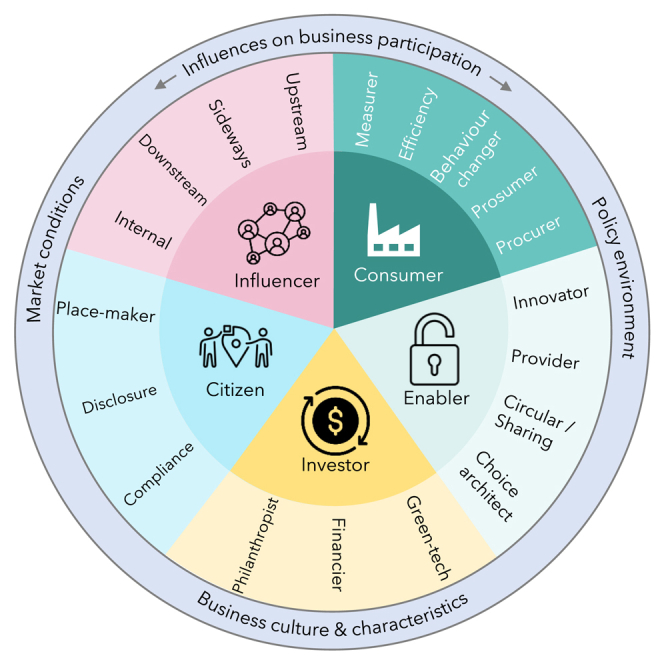

## Introduction

Businesses are at the center of the climate crisis. Through their activities they generate the majority of global greenhouse gas (GHG)^1^ emissions; yet their resources, innovation capacity and market reach mean they also hold unique leverage to drive decarbonization. Redirecting this influence toward net-zero pathways is one of the most urgent challenges of our time. They contribute approximately 70% of GHG emissions and could play a critical role in developing zero-carbon technologies and solutions which help accelerate sustainability transitions. Acting in partnership, businesses can play an even greater role in the governance of climate change. Coalitions such as the UN Global Compact and we mean business have galvanized the voice of business, calling for greater ambition from governments and the international community. However, a large proportion of the business community are not doing enough to decarbonize their own operations or contribute toward the wider social and economic change required to meet internationally agreed climate goals, notably the Paris agreement’s target of limiting global warming to well below 2°C and pursuing efforts to limit it to 1.5°C above pre-industrial levels.^2^

Barriers to climate action are well documented, with scholars identifying the voluntary nature of climate commitments and inadequate governance structures—as well as lack of knowledge among business leaders and limited access to finance—as among the most significant.^3^^,^^4^^,^^5^ However, it is also well established that corporations themselves can act as powerful barriers to climate action. Far from being a matter of ignorance, many firms—particularly in carbon-intensive sectors—have historically sought to delay, weaken, or shape climate policy and public understanding through lobbying, public relations, and the manufacture of doubt.^6^^,^^7^ There has also been increased scrutiny of corporate sustainability efforts, including the use of questionable carbon credits and offsetting claims,^8^^,^^9^ as regulators, civil society, and consumer groups are becoming active in exposing so-called “greenwashing”.^10^ In response, some businesses—even those considered leaders—are becoming more reticent about communicating their efforts (“greenhushing”), and others are even scaling back their climate activities.^11^ Greenhushing refers to the deliberate under-communication or withholding of information about a company’s sustainability initiatives, often to avoid public scrutiny, criticism, or accusations of greenwashing; unlike greenwashing, which exaggerates environmental claims, greenhushing can result in positive climate actions going unnoticed and may hinder transparency, industry learning, and wider progress on climate goals.^12^

Nonetheless, an increasing number businesses are quantifying their own emissions and setting reduction targets.^13^ Climate action is beginning to emerge as a mainstream business activity not just for large corporations and consumer brands, as many small- and medium-sized enterprises (SMEs are becoming more proactive).^14^ This trend is of vital significance, considering that SMEs represent around 90% of the business population across OECD countries, and produce roughly 50% of emissions from the business sector.^15^ There is an imperative to support these mainstreaming processes by providing clear, evidenced-based information to help all businesses to prioritize effective actions and capitalize on their unique capabilities. This article therefore addresses the question “what can businesses do to make a positive contribution to climate action?”.

While this article focuses on the constructive potential for business climate action, it is critical to acknowledge the extensive literature and evidence on businesses obstructing or delaying progress toward climate goals. Numerous studies have documented how some firms—especially those with vested interests in high-carbon sectors—exert influence on policymaking, voluntary governance schemes (such as SBTI and GHG protocol), and public discourse, with the deliberate aim of weakening or postponing climate regulation.^6^^,^^16^^,^^17^^,^^18^ These efforts include direct lobbying, strategic funding, and dissemination of doubt, representing significant barriers to effective climate action. A full assessment of the role of business in contributing to and addressing climate change is beyond the scope of this article, which is deliberately focused on the question of what business can do to take positive action in response to ongoing challenges—particularly given phenomena such as greenhushing, and the need among many businesses (especially SMEs) for practical guidance on making meaningful contributions. Since the research literature on business and the climate crisis is inherently interdisciplinary, spanning business studies, organizational psychology, economics, geography, sociology and more, it is difficult to navigate for researchers, let alone policymakers and business leaders. If climate action is to become mainstream, there is a need for a synthesis of this growing body of research, and for frameworks which resonate with an audience beyond the academic community. We respond to this challenge by developing a new framework for understanding the *multiple roles* that businesses can play in addressing the climate crisis. In this article, we make two primary contributions: first, a conceptual framework that synthesizes interdisciplinary theory on business roles in climate action; second, empirical analysis based on sectoral case studies, stakeholder interviews, and workshops. Taken together the framework deepens our understanding of businesses as potential climate actors, underlines roles that are often overlooked, and supports novel ways of framing policy challenges.

In the next section we discuss the methodology and process behind developing the framework over the course of a two-year research project. We then introduce the five roles, outlining key activities and sub-roles, and the barriers and opportunities which influence their adoption by business. Finally, we discuss the implications for business and policymakers, arguing that businesses must adopt a wider range of roles if climate action is to become mainstream and that policy is needed to create the drivers and incentives for doing so.

## Developing the multiple roles framework

This article is the result of a two-year research study on the governance of net zero for business *(GoZero)*. The project compared governance approaches internationally and within the UK, and selected five sectors to conduct in-depth empirical research: restaurants, construction trades, hairdressers, steel value-chain, and horticulture. These sectors were selected in a deliberative process which involved examining secondary data on their market size, emissions, as well as seeking coverage of a diverse challenges and opportunities with respect to climate action. For instance, while the steel sector is known to be energy-intensive, less known is that energy also represents a significant proportion of operational expenditure for restaurants and hairdressers, while some horticulture businesses are heavy consumers of natural gas (e.g., indoor tomato growers). Construction trades are vital providers of low-carbon technologies and building retrofit, while hairdressers are known for having conversations with clients, which can include discussions about climate change and sustainability.

The project involved a comprehensive policy review, along with interviews with 83 governance stakeholders and 70 businesses. Interview participants were recruited via purposive and snowball sampling, targeting individuals with sectoral expertize, organizational responsibility, or direct decision-making authority regarding climate responses. Sampling focused on the five sectors, although interviews were also conducted with national and sub-national governance stakeholders. Ethical approval for all activities was obtained from the University of Oxford’s Central University Research Ethics Committee (CUREC), code SOGE1A2021-242. Informed consent, encompassing withdrawal rights, was obtained prior to participation. Due to the risk of reidentification, raw data are not in the public domain.

This mixed-methods design, involving sectoral analysis, stakeholder interviews, and policy review, enabled methodological triangulation—enhancing the robustness and validity of our findings through integration of multiple data sources. Nonetheless, interview-based research is susceptible to limitations including restricted generalizability due to sectoral and geographical specificity, as well as selection, interpretive, and recall biases inherent in qualitative research. As such, this article synthesizes empirical findings with a comprehensive literature review to construct a novel analytical framework of business roles in climate action.

**Figure 1:**
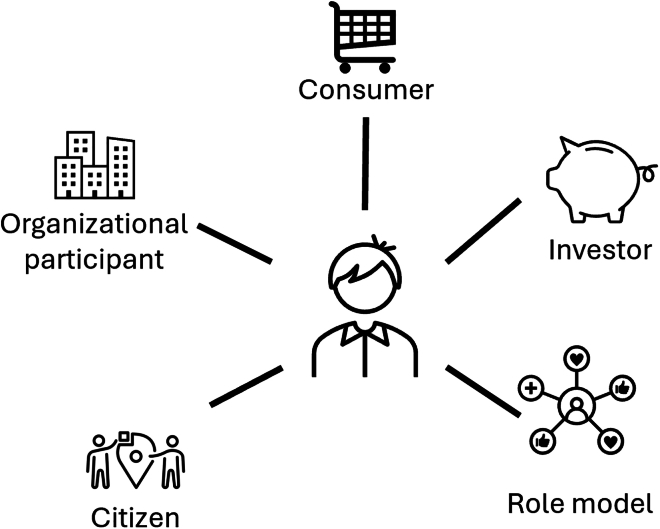
Five roles for high socio-economic status individuals, adapted from Nielsen et al.^20^

The development of our theoretical framework followed an abductive approach, which is an iterative process of moving between empirical observations and theoretical concepts to generate new insights.^19^ The framework is influenced by recent work exploring the multiple roles that individuals can adopt for climate action,^20^^,^^21^ and which is gaining influence in climate discourse and policy.^22^ Indeed, we used Nielsen et al.’s framework (see Figure 1) as a prompt in interviews to explore how climate policy and governance could better respond to businesses’ multiple capacities.

During *GoZero*, the several versions of the framework were developed and tested through stakeholder interviews, and in two deliberative workshops with researchers and business advisors in 2023, and in an industrial decarbonization panel at an international conference in 2024 (*reference omitted*). This abductive methodology was informed by the iterative application of theory to emerging empirical findings, as well as an ongoing review of academic and policy literature throughout the project.

Following the two workshops, the author team set out to conduct a systematic review of the literature to identify evidence on the multiple roles of business in climate action. However, it quickly became apparent that a traditional systematic review was not well suited to the topic. While systematic reviews can be expanded to include searches of gray literature using platforms, such as BASE and OpenGrey, our preliminary scoping revealed significant challenges. The diversity and inconsistency in how business roles are described across sectors, regions, and document types meant that developing a comprehensive set of search terms was impractical, and that even advanced gray literature tools would not reliably capture the breadth of relevant evidence. Much of the most pertinent insight is embedded in practitioner reports, policy documents, and industry publications that are poorly indexed or dispersed across isolated sources.

After consultation with bibliographic specialists, we concluded that any attempt at a fully systematic review, even with an expanded and inclusive search strategy, would risk missing key pro-climate business activities not readily identifiable using simple search terms—especially those relevant to SMEs. On the other hand, an iterative, abductive approach allowed the authors to explore evidence relating to particular roles using wide range of search terms and snowball sampling. We used major interdisciplinary databases including Scopus, Web of Science, Google Scholar to find relevant sources, supplemented by targeted Google searches for gray literature. Search terms combined combinations of “business climate action”, “roles”, “decarbonization”, and Perplexity.ai, an AI-powered search platform, was employed to identify sector-specific case studies, the accounts of which were then analyzed. Despite efforts for comprehensive coverage, the review inevitably prioritizes English-language scholarship and sources accessible via international databases, thereby presenting limitations of language bias and the risk of underrepresentation of regionally published or non-English research.

**Figure 2:**
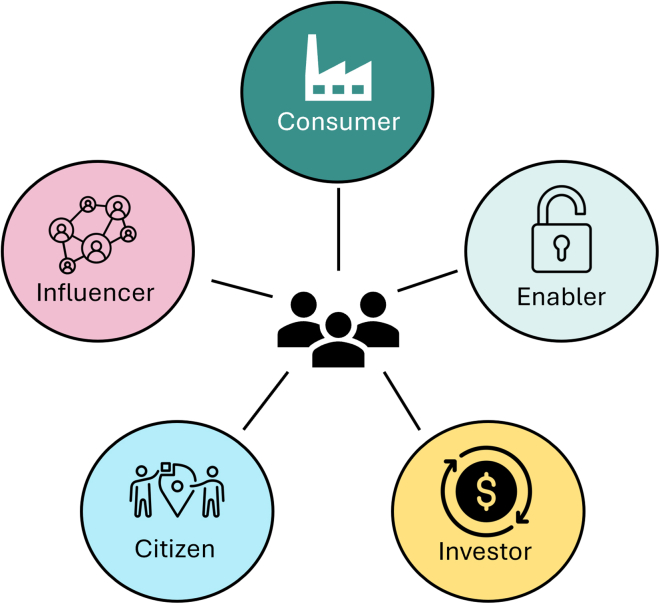
Multiple roles of business for climate action

The result is a structured narrative review which incorporates evidence from diverse academic, policy, and business sources, and integrates empirical findings and insights generated through *GoZero*. Figure 2 synthesizes insights from iterative empirical research and expert workshops conducted during *GoZero*, and visually presents the conceptual framework developed to illustrate the five diverse roles businesses can adopt for climate action. As with related frameworks such as Nielsen et al.,^20^ the framework is deliberately simple and conceptually accessible, intended to encourage broad engagement. However, beneath this simple visual lie a variety of subroles and detailed activities, which we elaborate upon in the following section.

## Five roles for business climate action

This section highlights trends, opportunities, and challenges facing businesses in adopting each role and sub-role, drawing on literature and empirical insights from *GoZero*.

### Consumer

The single most important way that most businesses can contribute positively to climate change mitigation is by reducing their direct GHG emissions from owned and controlled sources—for example by cutting fossil fuel use, investing in renewable generation, electrifying vehicle fleets and heating systems, and improving operational efficiency. In the EU, businesses consume around three-quarters of net domestic energy use,^23^ and while consumption is roughly proportional to business size, the collective use of energy by SMEs amounts to roughly half of emissions from the business sector.^15^

#### Measurement

Reducing the environmental impacts from consumption begins with measurement. The adage “you can’t manage what you don’t measure” underpins corporate environmental management,^24^ and yet only 11% of businesses in the UK measure their carbon footprint each year.^25^

Corporate GHG accounting is internationally standardized into scopes 1, 2, and 3,^26^ and many platforms exist to promote transparent reporting and target setting.^27^ While there is a plethora of tools and calculators available to help businesses calculate their impacts, uptake is limited, particularly among SMEs.^28^ Organizations such as the science based targets initiative and the SME Climate Hub have developed streamlined processes to adapt the relatively technical GHG protocol for use by smaller organizations, with minimal requirements such as optional scope 3 reporting. Yet there is criticism of the universal GHG protocol in academic literature, which is said to inadequately account for influenced-emissions and limit comparability.^29^^,^^30^ PepsiCo estimates that 92% of its emissions come from its value chain (scope 3), and most of its suppliers have no climate targets. The vast majority of impacts from many cosmetic products such as shampoo come from consumer use (hot water for showering).^31^ Some scholars argue that a system more closely aligned with financial accounting would enable more widespread environmental reporting^32^; while some lobby groups representing the financial sector are calling for using open banking protocols to automate emissions reporting for SMEs.^33^

In *GoZero*, businesses and industry stakeholders highlighted the challenges around data collection in many sectors, especially among businesses without advanced metering infrastructure. Restaurants’ largest share of emissions is derived from food (scope 3), for instance. Gathering data on quantities of ingredients used is challenging for many businesses, let alone finding appropriate and robust emissions-factors. Tellingly, in its framework for assessing sustainability, the sustainable restaurant association does not require applicants to quantify emissions.

#### Efficiency and behavior change

Optimizing the efficient use of energy and other resources is widely recognized to be the first priority for businesses seeking to reduce their environmental impacts, as these actions can also deliver cost-savings and productivity improvements.^34^ Extensive research on energy use in non-domestic buildings finds unrealized potential for measures such as fabric insulation, improved glazing, LED lighting, and behavioral measures, amounting to 10%–35% savings for a typical business premises.^35^^,^^36^^,^^37^ Analysis of 280 audits in Europe found 37% of all recommended energy saving actions involves zero-capital investment.^38^ Similar efficiency opportunities exist for process energy efficiency in manufacturing^34^ and vehicle fleets.^39^^,^^40^

Energy-intensive businesses (those with high energy-spend relative to revenue) are more likely to maximize efficiency.^41^ However, businesses often face barriers to implement efficiency measures, especially SMEs. *GoZero* interviews with stakeholders in the horticulture and steel sectors in the UK highlighted how SMEs such as (indoor) tomato growers and steel fabricators were highly exposed to the energy price crisis of 2021–2023, but were far less resilient than larger businesses in their ability to absorb, adapt, and bounce back.

As such, the so-called *efficiency gap* continues to persist.^34^ A substantial literature reports various barriers, ranging from access to finance, lack of awareness, short-term tenancy agreements and competing priorities.^36^^,^^42^^,^^43^ Energy service company (ESCo) business models have gained traction with larger, energy intensive businesses,^44^^,^^45^ but efforts to engage SMEs have been limited.^46^^,^^47^ Incentive policies have been implemented—largely in wealthier countries—to address the efficiency gap. Grants, loans, and subsidized audits are most commonly used,^3^^,^^37^ but other innovative examples include learning networks (see *influencer* role), and the Industrial Assessment Center scheme, which matches engineering graduate students with SMEs in the US, and has delivered over 20,000 building audits.^48^

#### Sustainable sourcing

Beyond efficiency, as businesses seek to eradicate emissions from their operations, sourcing of renewable energy becomes a priority. In the US, 72% of large businesses in 2016 reported buying renewable energy,^49^ and its voluntary green power market has seen strong growth, representing 6% of retail electricity sales in 2022.^50^ In the UK, while more than 30% of households purchase electricity on renewable tariffs,^51^ only 16% of small businesses do the same.^52^ However, 7% of UK businesses can be considered “prosumers”, having installed on-site renewable energy technologies such as rooftop solar, which is higher than the proportion of “prosumer” households (5%).^53^ Businesses are increasingly responding to fluctuating power prices from intermittent renewable supply, although SMEs lag behind larger industrial users.^54^

Barriers such as access to capital, short-term tenancy, and physical constraints limit the deployment of onsite renewables for many businesses.^55^^,^^56^^,^^57^ However, growing willingness to invest coupled with falling costs of photovoltaics and battery-storage systems are helping to drive uptake.^56^ A wider variety of shared ownership models also help overcome barriers to renewable deployment among businesses, especially when up-front cost is the foremost obstacle.^58^^,^^59^^,^^60^ Reduced battery costs also make solar-storage coupled systems more affordable, but a relatively limited range of electricity tariffs continues to hamper widespread engagement with demand response.^61^^,^^62^

For heating and transportation, fossil fuels continue to dominate business energy use, making electrification a priority for businesses. A prime example is heat pump (HP) technology, which uses electricity to provide heating and cooling, and when powered by renewable electricity, offers a highly efficient and low-carbon alternative to conventional fossil-fuel-based boilers and heaters. HPs are being deployed not only for space heating and hot water, but also in many industries using process heat such as food and beverage, materials production, and agriculture.^63^^,^^64^^,^^65^ Electrification of business vehicle fleets is underway, although progress with larger vehicles remains slow: electric truck sales represented less than 0.1% of total sales in the US in 2023.^66^ China is by far the market leader, with sales reaching 38,200 in 2023 (2.8%).

Rapid cost reductions also help accelerate vehicle electrification, and innovations in battery technology and design stimulate a wider range of commercial vehicle offerings.^66^ However, high up-front costs, inadequate charging infrastructure, and the need to alter operations or even business models are cited as key barriers preventing the widespread electrification of business fleets.^67^ Barriers to HP adoption are more intransigent. Learning rates are lower than for other low-carbon technologies,^68^ up-front costs are high, and installation often requires bespoke design, limiting scalability.^69^ Nonetheless, several countries have achieved significant penetration of HPs in both residential and non-residential buildings, indicating that policy enablers can achieve success. One factor that hampers the uptake of both EVs and HPs is the relative price of electricity and fossil fuels. In Sweden and the Netherlands the electricity-to-gas price ratio is less than 1.5, whereas in the UK and Belgium it has reached more than 5.^70^ This is also true for petrol and diesel in some markets,^71^ and there is an urgent need to remove fossil fuel subsidies in places such as Malaysia and Thailand, which impede business fleet electrification.

Lastly, businesses operating in supply chains can decrease their impact as consumers by sourcing and utilizing more sustainable resources. This includes the procurement of materials for processing, from raw mineral extraction through to manufacturing and distribution. Challenges related to supply-chain transparency have been well documented in the literature,^72^^,^^73^ but digitalization and the wider availability of data enable companies to make sustainability-informed procurement decisions.^74^ Table 1 summarizes the key sub-roles, activities and examples associated with the consumer role.

**Table 1:** Consumer and sub-roles

Main role	Sub-roles and activities	Example activities
Consumer	measurement and disclosure	calculating annual carbon footprint; reporting; target setting
	Efficiency	improving process and operational efficiency; fleet optimization; use of heat pumps
	pro-environmental behavior	energy management; employee engagement
	prosumer and demand-responder	on-site renewable generation; demand response
	sustainable procurement	PPAs; green electricity tariffs; supply-chain monitoring; sourcing sustainable materials

### Enabler

Businesses are the principal actors for developing, innovating, and diffusing the environmental goods and services which underpin sustainability transitions. As providers, innovators and choice architects, they can enable householders, other businesses and even governments to make low-carbon choices.

#### Innovation

While there is no doubt that the green economy is growing in almost every market,^75^ there are inherent difficulties in quantifying sustainable economic activity. The OECD’s^76^ framework includes production, consumption, and natural asset metrics, while the EU’s EGSS accounts for market and non-market outputs.^77^ The OECD distinguishes between *eco-innovators*, which develop new or improved products, services and processes which reduce environmental impact *with or without* intent; and *eco-entrepreneurs*, which tend to incorporate sustainability into the core goals of their entrepreneurship.^78^^,^^79^ Conventional examples include manufacturers of renewable energy technologies, energy efficient appliances, and electric vehicles, but as eco-innovation has proliferated there are myriad examples of green start-ups developing plastic alternatives^80^^,^^81^ and carbon capture and utilization.^82^

While there is a long history of popular literature discussing the characteristics of successful entrepreneurs,^83^^,^^84^ a more recent trend has been to investigate the necessary conditions for thriving innovation ecosystems.^85^^,^^86^ One review identifies key roles, such as “ecosystem leader”, “assembler”, and “champion” that are necessary to foster entrepreneurial innovation, and which can be played by different actors depending on context.^87^ In *GoZero*, we observed how many regions had identified growth in the green economy as an opportunity to build public-private partnerships, highlighting local strengths to pitch for government and business investment. City-regions around the world now compete with each other to attract eco-entrepreneurs and are indexed and ranked.^88^^,^^89^

#### Circularity and sharing

Circular business models (CBM) are increasingly promoted, through adoption remains uneven. The circular economy concept focuses on preserving natural resources, maximizing resource outputs, and eliminating negative externalities, such as emissions and pollution.^90^^,^^91^ Most definitions highlight the extension of product lifespan as a main component of CBMs, but circularity can also involve substituting products for services (servitization),^92^ such as media streaming, or mobility-*as*-a-service.^93^ Having attracted significant conceptual attention, there is a shift in the literature toward empirical studies of the implementation of various CBMs.^94^

Similarly seeking to reduce waste and over-consumption, businesses are also leading the expansion of the *sharing* economy. Made possible by the proliferation of internet connected devices and peer-to-peer infrastructures such as blockchain, the sharing economy has transformative potential.^95^^,^^96^ Car sharing is a typical example: since cars are idle 95% of the time, a sharing solution would significantly reduce the number of cars required.^97^ Sharing business models can also lead to the use of more durable materials and active recycling^98^ as well as offer cheaper access to services that help disadvantaged populations.^99^^,^^100^

Entrepreneurs in the circular and sharing economy face unique structural barriers, in seeking to overcome extractivism and the entrenched system of production and waste disposal.^101^^,^^102^^,^^103^ Repairing, sharing and repurposing items also requires changes to habits, behaviors and social norms.^104^^,^^105^ To unleash the potential for circular and sharing business models, structural change is needed, led by local and national governments in collaboration with business, social enterprises, and civil society. While innovations such as digital tools and even artificial intelligence can help,^106^ more substantive interventions include infrastructure investment, aggressive landfill taxes, and regulation of product design and single-use products.^103^^,^^107^^,^^108^ Interviews with companies in the steel industry highlighted myriad structural barriers to CBMs, from the technical difficulties of producing and recycling steel using low-carbon processes, to the extensive testing requirements associated with reusing steel in some contexts (e.g., construction), and perceived cultural resistance within the sector to innovation.

#### Middle actors and choice architects

While eco-innovators and eco-entrepreneurs attract most attention, there is a vast swathe of crucial “middle actors”, which distribute, install, maintain, and repair low carbon products and infrastructures.^109^ These include architects, building trades, mechanics, heating engineers, electricians, and plumbers: many of which fall outside conventional accounts of the green economy, but play a critical role in enabling the decarbonization of buildings and transportation systems.^110^^,^^111^ However, not all middle actors enable decarbonization; incumbent actors in sectors, such as utilities, construction, and aviation have sometimes resisted change or actively undermined policy and technological innovation due to vested interests or cultural inertia.^112^^,^^113^

More peripheral in the green economy literature are businesses which have unique opportunities to influence the consumption choices of customers. The good news is that a wide range of businesses can act as climate choice architects, through their engagements with customers, clients, and peers. “Nudge” interventions lend themselves to experimentation and incremental deployment, especially in online settings. This includes restaurants, grocers, and other retailers, which can adopt the role of “choice architects” in guiding customers to make lower-carbon choices.^114^ These interventions have been shown to be effective in food service settings,^115^^,^^116^ and include adjusting menu design,^117^ carbon labeling,^118^ providing and encouraging the use of “doggy bags”,^119^ and implementing meat-free default options.^120^^,^^121^ In the grocery sector, the last decade has seen substantial growth in the number of retail outlets offering reuse and refill solutions, although concentrated in Europe and North America.^122^

Large corporations have significant potential to influence consumer choice with relatively simple tweaks to choice architecture. In 2021, Google introduced a fuel-efficient routing feature into its Maps application, using a leaf icon to identify the greenest option. Google claims this reduced emissions by 1.2 MTCO2e in its first year.^123^ However, Maps also suggests to users that flying is a faster option than other modes, failing to account for the additional time taken for travel to airports, passport control, and security checks.^124^ Similarly, in the 2010s, Amazon introduced a “no rush shipping” option, rewarding customers with credit for selecting slower deliveries. This was removed in 2020, however, following investments which allowed the corporation to increase delivery capacity.^125^ While Amazon claims its deliveries are more environmentally friendly than customers driving to stores,^126^ there are clear opportunities for further efficiencies to reduce its large carbon footprint. These examples highlight the potential impact of nudges, which remain under-utilized. The imperative to innovate in this way is especially necessary given large recent increases in emissions from major technology firms, such as Google^127^ and Amazon,^128^ largely driven by the energy demands of expanding artificial intelligence capabilities.

The conditions which enable middle actors to fulfill their potential to be climate leaders are better developed in some countries than others. In Germany, there are established governance promoting the success of the so-called *Mittelstand* (medium-sized businesses), alongside recognition of their vital role in the energy transition.^129^ Here, building-related trades are well-regulated and valued for their unique skills.^130^ By contrast, evidence from *GoZero* found that construction and related trades in the UK are inhibited by an aging and largely male workforce, which lacks the skills required for designing and installing energy efficiency and renewable energy solutions.^131^^,^^132^^,^^133^ Worse, some incumbents such as gas-heating engineers and car mechanics have a vested interest in downplaying the benefits of alternative technologies such as heat pumps and electric vehicles.^17^ Such micro-politics can have large-scale ramifications. Recently, the UK government reversed plans to fine boiler manufacturers if they failed to meet minimum quotas for installing heat pumps, in response to emerging evidence that such businesses intended to pass the cost of fines on to consumers.^134^ Providing training opportunities for incumbents, boosting diversity, and raising standards for qualifications and accreditations can help to realize the potential of middle actors. Closing the green skills gap is becoming a higher priority across major economies.^135^ Table 2 summarizes the four sub-roles discussed here.

**Table 2:** Enabler and sub-roles

Main role	Sub-roles and activities	Example activities
Enabler	eco-innovator and eco-entrepreneur	developing new zero-carbon technologies and solutions, e.g., renewable energy, appliances, plastic-alternatives, CCUS
	Provider	designing, maintaining and repairing infrastructures including buildings and vehicles
	circular and sharing economy business models	design-for-repair; reuse; servitization; shared-ownership; peer-to-peer trading
	choice architect	setting low-carbon defaults; menu design; highlight eco-options

### Influencer

**Figure 3:**
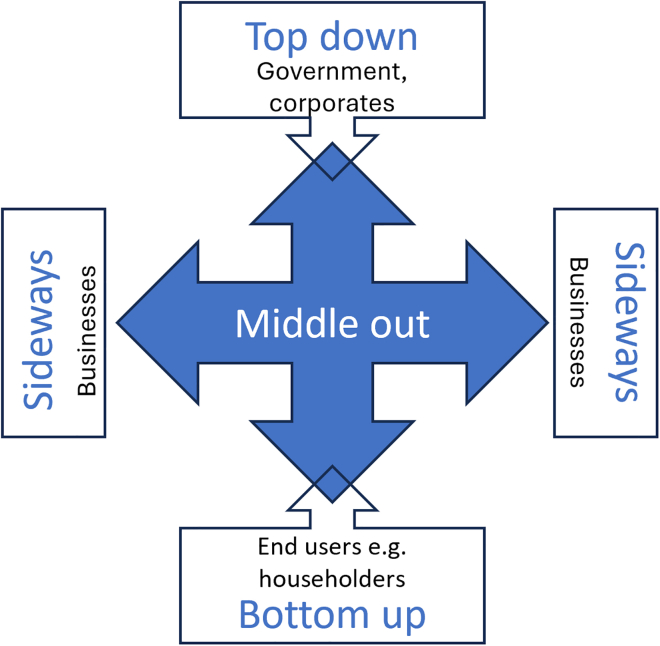
Modes of influence, from the middle out perspective (based on Parag and Janda, 2014)

Businesses are socially and culturally embedded actors that create, consolidate, and sometimes disrupt norms. Their influence extends into every part of social, political, and economic life, with the ability to effect consumption norms, policy decisions, trade patterns, and workplace behavior. Here, we employ the middle-out perspective framework (See in the study by Parag et al.,^109^ and reproduced in Figure 3), categorizing these forms of influence as downstream (publics), upstream (policy makers), sideways (other businesses), and add an internal orientation (employees). These sub-roles are summarized, with examples, in Table 3.

**Table 3:** Influencer and sub-roles

Main role	Sub-roles and activities	Example activities
Influencer	upstream	lobbying; providing evidence to policymakers
	sideways	networking; peer-to-peer learning; B2B services
	downstream	dialogue; community engagement
	internal	green teams and champions; training; gamification; incentives

#### Upstream

Acting *upstream*, businesses can influence governments and international bodies directly, for instance by responding to consultations, giving evidence, and lobbying.^136^ The business delegation at international climate negotiations has grown substantially in recent years.^137^^,^^138^ While this has attracted criticism by commentators observing the increasing representation of fossil fuel interests,^139^^,^^140^ conversely, UNFCCC conferences have also provided a platform for business leaders to form ambitious coalitions. The We Mean Business coalition was founded at COP21, representing progressive business voices and exerting pressure on governments and international bodies to strive for more ambitious climate goals. In advance of COP26, the SME Climate Hub and UN Race to Zero initiative actively encouraged businesses to make net-zero pledges. *InfluenceMap* is an initiative which analyses how companies exert their influence on the climate crisis, both in support and against policy action.^141^

While corporations and business lobbyists have conventionally sought to minimize the regulatory burden associated with climate action,^142^ this may be beginning to change. Our interviews with business representative organizations (BROs) in the UK revealed a growing recognition of the need for more climate regulation. Instead of opposing proposals for environmental obligations, they increasingly lobby for policies which provide investment certainty; and create a level-playing field which reflects the different capabilities of businesses. However, we also observed a tendency among BROs toward conservative approaches on climate action, as they must accommodate viewpoints from a wide range of smaller businesses. Too often the default position is to lobby for blanket exemptions for SMEs to environmental regulation, which serves to delay decarbonization progress.

#### Sideways

Businesses hold significant *sideways* influence too. Businesses interact in myriad ways through supply chains, local networks, online communities, and by utilizing professional services. There is little evidence on which sources of support are most valued by businesses for advice on emissions reduction, but a consistent finding from European SMEs surveys is that accountants and other business leaders are most trusted.^143^^,^^144^ Outside the EU evidence is limited, but one study investigating environmental business support in South-East Asia found that SMEs were most likely to seek government sources for advice.^145^

Supply chain engagement on emissions reporting and reduction is a growing activity, as corporations seek to reduce scope 3 emissions.^146^^,^^147^ IKEA, for example, developed a protocol and associated software platform for specifying environmental and labor standards for suppliers.^148^ Learning energy efficiency networks (LEEN) exemplify how sideways business influence can contribute to reduced emissions. Regional energy agencies and municipalities in Germany and Sweden recruit businesses—typically manufacturers—into cohorts to share knowledge on energy efficiency and carbon-saving initiatives, supported with subsidies and expert advice.^149^^,^^150^ While their success for emissions reduction is well documented, networks struggle for financial sustainability based on membership fees alone, and most successful examples are those supported by public funds through energy agencies, universities, or local authorities.^151^^,^^152^

Business-to-business (B2B) services for carbon reduction are becoming more widespread. Whereas the market for providing sustainability consultancy to large corporations is well-established,^153^^,^^154^ few consultancies have sought to target SME clients, given their lower capacity for expenditure on sustainability services, and the availability of public subsidies for energy audits or decarbonization advice.^37^^,^^155^ In *GoZero*, we charted the emergence of a new market niche, where consultancies increasingly target SMEs with subscription models featuring online calculators and emissions-savings advice. What has long been considered a market failure may thus be beginning to function. This has implications for policymakers, who must avoid using public funds to *crowd out* private offerings, but instead seek to reach SMEs not served by these consultancy services.

#### Downstream

Influence on customers and other publics occur in various ways. As businesses respond to consumer demand for sustainable products and services, they are increasingly using advertising and marketing to promote their environmental credentials. “Green marketing” can help promote environmentally friendly products, and consumers are more loyal to businesses perceived to be sustainable.^156^ However, consumers’ ability to distinguish the validity and accuracy of environmental claims is limited,^157^ and a “green gap” exists between consumers’ stated environmental intentions and their behavior.^158^ *Greenwashing* is attracting greater scrutiny from consumer groups and even regulators,^159^ and the notion of *Greenhushing* is being observed as some businesses are becoming reluctant to communicate their sustainability endeavors.^11^

Businesses also play a key role in addressing the problem of “pluralistic ignorance”, whereby people underestimate others’ climate concern and their willingness to take action.^160^ They can do this by using their interactions with clients, customers, and communities to help promote climate change in public discourse. Hairdressers, for instance, are renowned for conversing with their clients. In *GoZero*, interviews with sustainable salons, mainly Green Salon Collective members, revealed how hairdressers were incorporating climate change and sustainability into their client conversations. These conversations can be prompted in several ways, from current events to salon actions and clients’ interests, with hairdressers having the skills to adapt their approach depending on the type of client. The trust and relationships they have with clients, as well as how salons act as a social space, play a key role in how they navigate these conversations and can influence clients. “Mirror Talkers” used QR codes stuck on hair salon mirrors to stimulate discussions about sustainable hair care.^161^ Similarly, cafes and coffee shops in Canada, Australia, and the UK volunteer host gatherings for climate conversations,^162^ while the Sustainable Restaurant Association encourages its members to engage customers as part of their accreditation process. In 2022, staff in a Burger King outlet in Vienna, Austria experimented by asking customers “*normal*, or with meat?” in an effort to raise awareness of the impacts from meat and stimulate climate conversations.^163^

#### Internal

Businesses have significant influence over their own employees. The use of green champions and green teams within organizations has a long history, largely focused on identifying opportunities for energy savings and other efficiency improvements.^164^^,^^165^ A more recent trend is to provide environmental training for staff. The carbon literacy project has trained over 56,000 people in the UK, while the Climate School in France provides nearly 200 modules specifically for corporate employees. New service providers, such as *JouleBug* and *Pawprint* are helping corporates to engage staff on climate, providing behavioral nudges and gamifying sustainability, often alongside incentives for making low carbon choices, such as switching to active travel (e.g., *Climate Perks*). In *GoZero*, the values and motivations of hair salon owner/managers underpinned efforts to implement sustainability initiatives among staff, though this was not always successful. Workplace behavior change can also inspire green choices outside the workplace. However, there remains relatively little evidence on work-to-home behavioral spillover.^166^

All employing businesses can engage their staff on climate change, and face relatively few barriers to doing so. Employees and jobseekers are increasingly demanding that businesses are proactive on climate.^167^^,^^168^ Younger people especially are seeking climate leadership from employers.

### Citizen

There is both an expectation and demand that companies assume responsibility for how they impact the communities they operate in.^169^^,^^170^^,^^171^ A vast literature concerns organizational citizenship for the environment, which can be defined as “individual and discretionary social behaviors that are not explicitly recognized by the formal reward system and that contribute to a more effective environmental management by organizations”.^172^

#### Compliance and disclosure

Complying with environmental laws, regulations, and voluntary agreements is a basic requirement for corporate climate citizenship. The World Business Council for Sustainable Development (WBCSD), which tracks environmental regulations around the world, has identified a 7-fold increase in environmental regulations since the turn of the century. While evidence on the costs and benefits of compliance is mixed, much of the literature emphasizes boosts to productivity and environmental outcomes, supporting the “porter hypothesis”.^173^^,^^174^ Similarly, several studies document a positive relationship between disclosure and financial impact.^175^^,^^176^ Open reporting can provide stakeholders with essential data that help inform their decisions about interactions with the business, and increase businesses’ legitimacy and access to labor and financial capital.^177^^,^^178^ Accreditation standards such as B-Corp, ISO 14000, or Carbon Trust Certified can further reinforce businesses’ motivation.^179^

Where regulations do not require strict compliance, it can be difficult for businesses and their stakeholders to define and agree the extent of these responsibilities. As discussed previously, the GHG protocol has its critics, and there are moves to increase pressure on businesses to calculate and disclose their “influenced emissions”. Advertised emissions, which claims that advertising is responsible for over 200 Mt of carbon emissions in the UK alone (2021), is an initiative seeking to influence the advertising industry to take responsibility for their impacts on the emissions associated with increased consumption.

#### Community

Businesses’ operations influence both their institutional and physical surroundings. They can directly influence their community by reducing waste, conserving energy, sourcing materials responsibly, and supporting local environmental initiatives.^180^^,^^181^ Businesses installing workplace EV chargers can help promote uptake by staff, for instance, and installing rooftop solar can drive local uptake via the “solar contagion’” effect.^182^

The growing recognition of the role of businesses and the importance of community-based climate action has led to increased availability of grants, subsidies, and other financial support for businesses willing to engage as local climate actors.^183^^,^^184^ However, these activities do not necessarily require public money. In *GoZero*, we identified several forms of corporate citizenship focused on local capacity building. Area-based insetting (ABI) is one such example being trialled in Oxfordshire. Where corporations have committed to carbon neutrality, the norm is to use offsets from international carbon credits. ABI seeks to channel corporate investment into local carbon-reduction initiatives including those led by SMEs and non-profits, boosting social capital, and community benefit.

#### Governance

In the last two decades, academic literature has observed a shift from *government* to *governance* with respect to environmental issues.^185^ Businesses are increasingly able to contribute toward governance processes from the local to the supranational, including promoting climate action, public health, education, social justice, and human rights outcomes, or engaging in self-regulation to fill the gaps in legal regulation.^186^ Business influence is not necessarily aligned with climate action, however. Political resistance from fossil fuel companies in the US continues to hinder effective climate policy.^187^

In the UK, Project Perseus involves a transformative proposal to automate emissions reporting for every SME in the UK, addressing the lack of quality data and effective management of emissions among smaller businesses. The project leverages the open banking protocol to identify energy expenditure and calculate scope 1 and 2 emissions. Led by industry collective Bankers for Net Zero, this is an example of business-led innovation for climate governance.

Quantifying the benefits of being a good corporate citizen, such as reduced environmental impact, enhanced community resilience, or improved social capital, is often not captured in traditional business models or metrics.^188^^,^^189^ Especially among SMEs, skills gaps and/or lack of capacity pertaining to impact measurement and reporting can also make it more difficult to navigate policy regulations and community expectations.^190^

The four sub-roles associated with business climate citizenship are summarized in Table 4.

**Table 4:** Citizen and sub-roles

Main role	Sub-roles	Example activities
Citizen	compliance	emissions trading
	disclosure	monitoring and reporting
	community member	leading by example; convening
	governance actor	supporting community climate action

### Investor

Addressing climate change will require an increased flow of climate finance, improved climate finance governance, and a transformation of global financial systems.^191^^,^^192^ The total global flow of climate finance in 2020 amounted to USD 640 billion, which the IPCC warns must increase by 3–6× to meet the Paris agreement goals.^1^ In comparison, total subsidies for fossil fuels was USD 7 trillion.^193^ In the United States, fossil fuel, utility, and transportation sectors spent over USD 2 billion on climate-related lobbying between 2000 and 2016—vastly outspending environmental advocates and reflecting a concerted effort to delay or weaken climate policy.^18^ Such figures highlight the scale of financial resources mobilized to obstruct or slow climate action, in contrast to the levels of finance needed to achieve global climate goals. Despite these realities and reflecting this article’s focus, the following section focuses on the positive investments businesses can make to accelerate the low-carbon transition and deliver social and environmental returns.

#### Direct investment and financing

Investment underpins the multiple roles of business so far discussed. This involves not only direct investments in green projects, but also instruments such as green bonds, climate funds, blended-finance, and sustainability-linked loans that increase firm value, boost social and environmental performance, and hedge against climate risks.^191^^,^^194^^,^^195^ It is not only financial institutions that can act. In 2016 Apple issued a green bond at USD 1.5 billion to support sustainable projects across its supply chain.^196^ Microsoft has pledged to become carbon negative by 2030 and to remove all historical emissions by 2050, building a portfolio of projects across renewable energy, carbon removal, and innovation funding worth billions of US dollars.^197^

SMEs have conventionally relied on public finance for environmental improvements,^37^ despite compelling evidence of the business case for energy efficiency investments.^198^ Now, more private banks and financiers are developing green products targeted at SMEs, alongside advice and guidance for decarbonization.^199^ Evidence for SME demand for private finance remains weak, however, and there is a need for more research on the appeal and efficacy of different financial products.

Finance is crucial for adaptation as well as mitigation. The UN estimates the costs of adaptation to be $240 b/year this decade, and the current adaptation finance gap $194–366 b/year.^200^ Businesses play a pivotal role in closing this gap by investing in projects that build resilience to climate impacts.^201^^,^^202^ Importantly, given dangers such as increasing debt burdens and dependencies, business investments must be compatible with local ownership, civil society participation, and transparency.^203^^,^^204^

#### Philanthropy

Philanthropic investments are increasingly being channeled into climate action. By leveraging their financial resources and engaging as philanthropists, businesses can deliberately use their ventures to serve the public good and act as proactive agents of change.^205^^,^^206^^,^^207^ Momentum has surged for nature-based solutions, which address sustainability challenges while providing biodiversity and human well-being co-benefits (e.g., establishing or preserving urban green spaces, mangroves, or wetlands).^208^^,^^209^ For example, the $10b Bezos Earth Fund invests in renewable energy, sustainable agriculture, and climate justice. Still, philanthropic activity is spatially uneven. California hosts many of the largest philanthropic foundations, and benefits substantially from their investments.^210^ The decentralized nature of climate finance can lead to fragmented approaches that could undermine the overall efficacy of climate interventions.^211^ Complex and often inconsistent regulatory environments create uncertainty, which can make it difficult for businesses to compare investment opportunities, assess risks accurately, and ensure that investments are delivering the intended environmental and social benefits.^191^^,^^195^

To unlock the potential of businesses as investors for climate action, more coordinated efforts are required to encourage investments in long-term, sustainable initiatives. Aligning investment strategies with broader climate and sustainability goals—including integrating financial performance with environmental and social metrics—can help mitigate risks and enhance the positive impacts of private sector contributions.

Table 5 summarizes the ways in which business can act as pro-climate investors.

**Table 5:** Investor and sub-roles

Main role	Sub-roles and activities	Example activities
Investor	direct investment in green technologies	building energy management; building fabric upgrades; fleet electrification; renewable generation; research and development
	Financier	lending to enable businesses, communities and households to invest in low-carbon solutions; area-based insetting
	Philanthropist	donating to fill funding gaps; de-risking climate finance through blended models.

**Figure 4:**
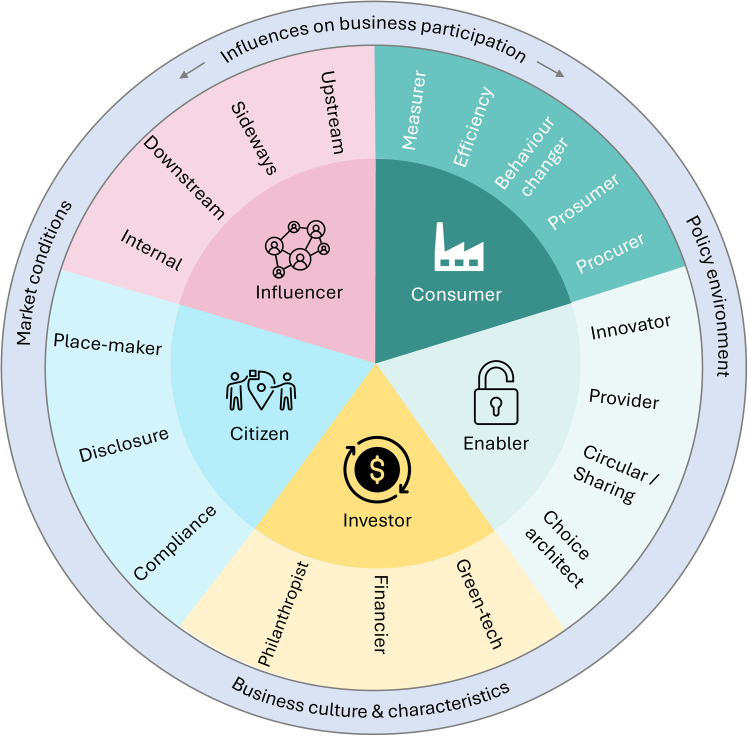
The five roles (inner circle) and 18 sub-roles and activities (middle circle) identified for businesses to take positive climate action The outer circle demonstrates key influences on businesses’ ability to assume the roles.

The preceding sections have outlined both the conceptual framework and the empirical evidence for each role businesses can play in climate action. Figure 4 builds on the five-role framework by incorporating the variety of sub-roles discussed in the previous section, and highlighting *policy environment*, *market conditions*, and *business characteristics* as important variables that influence business participation in different forms of climate action. Additionally, to aid comparison and synthesis, Table 6 summarizes the key barriers and opportunities that influence the adoption of each role. This overview distils findings from across different sectors, highlighting actionable insights and persistent challenges, and serves as a foundation for the discussion that follows.

**Table 6:** Summary of key barriers and opportunities for businesses adopting multiple roles for climate action

Role	Key barriers	Key opportunities
Consumer	- limited access to sustainable products/services - higher costs associated with sustainable choices - lack of awareness or information on sustainable alternatives	- growing demand for green products/services - potential for cost savings through efficiency and reduced waste - ability to influence market through responsible purchasing decisions and reporting
Enabler	- resource constraints (e.g., financial, technical expertise) - resistance to change within value chains - lack of supportive policy frameworks	- potential to drive innovation in sustainability solutions - collaborative platforms for sharing best practices - emerging support for sustainable supply chain initiatives
Influencer	- limited influence in policy-making and public discourse - resource constraints for sustainability communication - lack of platforms for effective engagement with stakeholders	- ability to shape public opinion and consumer behavior through advocacy - leadership in industry sustainability standards - growing media and digital platforms for business voices
Citizen	- perceived high costs of sustainable community engagement - challenges in responsibility/measuring impact of citizenship activities - limited resources for philanthropic or community-based actions	- building social capital and community trust - enhanced brand reputation through community-focused initiatives - access to partnerships with local governments and NGOs - business internal/ownership structure
Investor	- limited access to green finance and investment capital - uncertainty around returns from sustainability investments - regulatory and market uncertainty	- opportunities in impact investing and green bonds - potential for risk mitigation and long-term savings - growing investor demand for sustainable business models

## Discussion and conclusion

This article has developed a novel framework for understanding the different ways businesses are implicated in climate action, drawing on illustrative empirical research from the UK. Our framework seeks to produce a more expansive discourse about the role of business in climate action. While direct emissions reduction remains the most urgent and important step that businesses of all sizes can take, this article also highlights how businesses can accelerate climate action in other ways, such as by enabling and influencing others’ actions, acting as a climate citizen and investing in climate solutions. This provides a new perspective on businesses as climate actors, which opens avenues for scholarship as well as for societal understanding of business roles. This framework helps to overcome unhelpful dialectics about businesses as climate “villains” or “heroes”, and potentially inspires businesses to think about the variety of ways they can make a difference.

To achieve climate goals, there is an urgent need for more businesses to adopt the roles outlined previously, and for greater policy and societal pressure on them to do so. The framework introduced here is deliberately intended to be universal, such that no business should be *unable* to adopt each role. However, businesses have different capabilities, and a range of factors enable and constrain their ability to implement pro-environmental behaviors. It is important to acknowledge the different contexts in which business climate action takes place, and the key factors (highlighted in the outer circle in Figure 4) that shape what is possible or desirable for individual businesses. Here, we analysis the challenges and opportunities relating to these factors within differing contexts.

Firstly, *market conditions* vary significantly across sectors and have an important bearing on the roles that businesses might perform and how they perform those roles. For example, consumer-facing sectors have greater opportunities to directly influence end-user behaviors, as highlighted in the case of hairdressers above. This is not universally true, however. While restaurants have direct contact with customers, our research found that restaurateurs and chefs recognize the “treat” function of a meal out, which impacts how they assess what their customers want on menus. This sometimes leads to a reliance on red meat, which is often perceived to be something that clients would not cook at home, and thus desire in restaurants. Innovation with lower-carbon options, e.g., venison or meat-free dishes requires additional skills and ingredients which may be more difficult to source, while also incurring risk.

Business to business sectors (such as steel supply chains) are less likely to have direct impact on consumer behavior and operate interdependently with a range of other businesses. Their position within supply chains might however provide opportunities to develop partnerships to influence change across supply chains, especially if working with “Tier 1” suppliers. Similarly, those operating in integrated supply chains might face challenges to acting as innovators when specific standards and methods have been agreed between supply chain actors, or as industry standards. Dynamics within supply chains are also important: businesses operating within more fragmented, cost-competitive supply chains are likely to find it harder to influence other supply chain businesses than in more settled, trust-based relationships, or those who already hold a dominant position within supply chains. Market conditions will also impact access to resources for action. For instance, capital intensive sectors (like steel businesses) require greater upfront investment but could also have greater access to finance and government initiatives, whereas those operating in low-margin sectors (like hospitality) might find it harder to finance upfront cost of retrofit or energy efficient equipment.

Second, differences in *policy and regulatory environments* between sectors and places also matters. Policy signals vary significantly and set the general tone for action. As a high-emissions sector, the steel industry is subject to growing policy and regulatory focus such as export measures like carbon-border adjustment mechanisms being introduced in the EU and UK. This sharpens focus on emissions reductions, which can spread—via the role of influencer—along supply chains. For climate investors, stringent regulation and fiscal incentives might be important, but policy stability is critical to making long-term decisions. Sectors with less regulatory focus are more likely to rely on self-governance, where climate leaders and standards organizations and other NGOs seek to raise the bar for sustainability practices.

Third, the *cultures and characteristics* unique to each business have a significant bearing on its climate capabilities. For smaller businesses, owner-managers values, knowledge and motivation are key determinants of climate action,^212^ whereas for larger businesses, management structures and organizational cultures are particularly important.^213^ More outward facing “extravert” businesses with broad networks might be well placed to act as influencers with other businesses and potential within their local communities. In contrast, “introvert” businesses might be less well placed to reach lots of other firms or people, but instead be able to utilize the smaller numbers of tighter connections with other businesses or local community to more deeply influence actions and behaviors among that group.

Finally, in bringing together our findings we underline three points of significance for the study and implementation of climate action among the business community.

(1) Adopting a multiple roles framing deepens understanding of businesses as important “midstream” actors,^214^ with potential to shape the choices and actions of a range of other organizations and individuals. Explicitly outlining these diverse roles allows us to identify where these roles might have most *salience*, and what *challenges* need to be unlocked to achieve their potential. Similarly, recent attention to climate intermediaries,^215^ including the “middle-out” perspective^109^ have emphasized the importance of knowledge brokers and solution providers in climate action. Our framework has potential to expand these conceptual approaches through broader consideration of intermediation in different forms.
(2) By labeling and unpacking different forms of business climate action we also draw attention to areas which receive less focus or are perceived as peripheral activities, such as forms of climate citizenship and influence. The multiple roles framework helps to mainstream *public-sphere* climate action by businesses, moving beyond the narrow focus of scope 1, 2, and 3 emissions. In doing so, it can derisk the pursuit of less tangible activities by business, providing a framework to highlight the importance of corporate climate citizenship and resist accusations of greenwashing. Policymakers may also be encouraged to facilitate different types of climate action in business. More effective and widespread action can occur if businesses both better understand the different ways they can make a difference, and are incentivized to do so.
(3) Following the aforementioned, adopting a multiple roles framework in policy can support framings of policy challenges, and in turn provide focus on where policy might be best placed to deliver interventions that unlock potential in different roles. Exploring interventions which best enable businesses to do so warrant further research and experimentation. For instance, how could policy action catalyze the role of customer-focused businesses as climate influencers, or place-based businesses as community citizens? Or how can policy to support action on businesses emissions are tailored to also support engagement with workers?

Despite the strengths of this study, several limitations should be acknowledged. Empirical data are predominantly derived from UK-based sectors and stakeholders, which may limit the generalizability of certain findings and nuances to other geographic and institutional contexts. While the conceptual framework is intended to be universal, its transferability to other countries, industries or regulatory environments warrants further empirical validation.

Future research might examine potential tensions or trade-offs between roles: for instance, how businesses balance the imperatives of decarbonizing their own operations and influencing others, or choosing where to place climate citizenship efforts (e.g., locally, nationally). Research may also explore how the pursuit of one role may constrain or enhance the potential for engagement in another. Further work could also explore how the framework might be operationalized—potentially through the development of indicators or practical tools—to support policy design and business strategy in diverse contexts.

In conclusion, this article has sought to open up a broader, more productive discussion about businesses might act as climate actors who connect with the world around them in myriad ways, and how they might use those connections to mainstream climate action. It seeks to mainstream conversations about the *potential* for business action (and the barriers that need to be addressed to unlock more action), and business climate *action itself* through inspiring more holistic views on the role of business for bringing about change. We hope this contribution will go some way toward countering a concerning tendency for *greenhushing* by pointing to a broader range of roles and actions that businesses can and should adopt, so long as they are transparent, deliberative, and consultative. However, the accelerating pace and severity of climate breakdown means that we cannot afford to wait for businesses to act at their own pace. Effective policy, regulation, and public pressure are essential to steer and accelerate the transition, ensuring that corporate climate action matches the scale and urgency of the crisis.

## Acknowledgments

This research has been enabled by funding from the 10.13039/501100000326UK Energy Research Centre Phase 4 Program (grant ref: EP/S029575/1).

This research involved interviews with human subjects. Informed consent was obtained from all participants, and ethical approval was granted by University of Oxford’s Central University Research Ethics Committee (CUREC), code SOGE1A2021-242.

We are not able to share data from the interviews undertaken as part of the GoZero project due to the risk of reidentification.

## Declaration of interests

The authors declare no competing interests.
